# Erratum to: Complete genome sequence of *Pirellula staleyi* type strain (ATCC 27377^T^)

**DOI:** 10.4056/sigs.881234

**Published:** 2010-05-15

**Authors:** Alicia Clum, Brian J. Tindall, Johannes Sikorski, Natalia Ivanova, Konstantinos Mavromatis, Susan Lucas, Tijana Glavina Del Rio, Matt Nolan, Feng Chen, Hope Tice, Sam Pitluck, Jan-Fang Cheng, Olga Chertkov, Thomas Brettin, Cliff Han, John C. Detter, Cheryl Kuske, David Bruce, Lynne Goodwin, Galina Ovchinikova, Amrita Pati, Natalia Mikhailova, Amy Chen, Krishna Palaniappan, Miriam Land, Loren Hauser, Yun-Juan Chang, Cynthia D. Jeffries, Patrick Chain, Manfred Rohde, Markus Göker, Jim Bristow, Jonathan A. Eisen, Victor Markowitz, Philip Hugenholtz, Nikos C. Kyrpides, Hans-Peter Klenk, Alla Lapidus

**Affiliations:** 1 DOE Joint Genome Institute, Walnut Creek, California, USA; 2 DSMZ – German Collection of Microorganisms and Cell Cultures GmbH, Braunschweig, Germany; 3 Los Alamos National Laboratory, Bioscience Division, Los Alamos, New Mexico, USA; 4 Biological Data Management and Technology Center, Lawrence Berkeley National Laboratory, Berkeley, California, USA; 5 Oak Ridge National Laboratory, Oak Ridge, Tennessee, USA; 6 HZI – Helmholtz Centre for Infection Research, Braunschweig, Germany; 7 University of California Davis Genome Center, Davis, California, USA

## Correction to figure 1

Volume 1 No. 3, p. 310, Figure 1 should appear as shown below. The mature cell shape has previously been described as teardrop- to pear-shaped, with the attachment pole slightly pointed [1]. Our paper showed a rod-shaped cell, which is not the correct morphology for this species.

**Figure 1:**
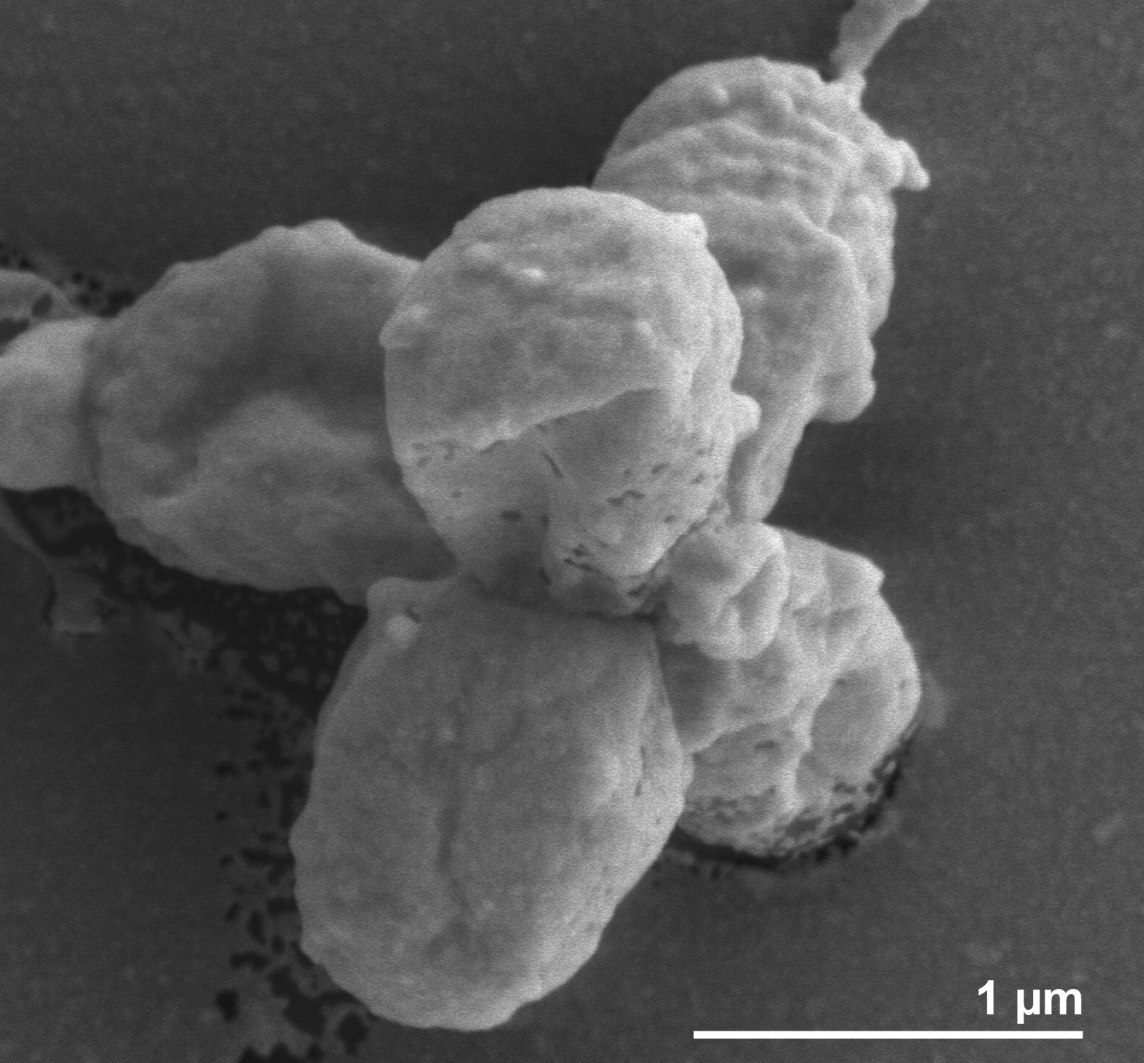
Scanning electron micrograph of *P. staleyi* ATCC 27377^T^
